# Potential synergistic effect of *Alhagi graecorum* ethanolic extract with two conventional food preservatives against some foodborne pathogens

**DOI:** 10.1007/s00203-022-03302-0

**Published:** 2022-11-01

**Authors:** Abdulrhman S. Shaker, Diaa A. Marrez, Mohamed A. Ali, Hayam M. Fathy

**Affiliations:** 1 Microbiology Department, Faculty of Agriculture, Cairo University, Giza, Egypt; 2 Food Toxicology and Contaminants Department, National Research Centre, Dokki, Giza Egypt

**Keywords:** *Alhagi graecorum*, Synergistic effect, Food preservatives, Molecular docking

## Abstract

The present study aims to screen the anti-bacterial activity and synergistic interaction of *A. graecorum* Boiss. ethanolic extract with two food preservatives against five strains of foodborne bacteria. Disk diffusion and minimum inhibitory concentration were used for anti-bacterial assay, checkerboard assay and time-kill curve were used for the combination studies. HPLC analysis and molecular docking study were performed to corroborate the in vitro results. The ethanolic extract showed anti-bacterial activity against all tested bacterial strains with inhibition zones from 7.5 to 9.3 mm and MIC values ranged between 1.2 and 1.8 mg mL^−1^. The combination of the ethanolic extract with Na-benzoate or Na-propionate resulted in synergistic and additive interactions against the tested bacteria with fractional inhibitory concentration index (FICI) ranges 0.31–0.63 and no antagonism was shown. Time-kill curve assay showed that the synergistic and additive combinations have inhibitory effects on the tested strains. The ethanolic extract combination with Na-benzoate or Na-propionate can be used for development new sources of food preservatives. Testing new different natural plant extracts with food preservatives will help develop new anti-bacterial agents.

## Introduction

Food and Drug Administration (FDA) estimates that two to three percent of all foodborne illnesses lead to serious secondary long-term illnesses. For example, certain strains of *E. coli* can cause kidney failure in young children and infants, *Salmonella* can lead to arthritis inflammation and serious infections, *Listeria* can cause meningitis and *Campylobacter* may cause Guillain–Barre syndrome (Oliver 2019). Sources of food contamination may be chemical or through infectious agents, such as bacteria, fungi, viruses and parasites. Consumption of contaminated food leads to foodborne illnesses, which in turn can lead to death. For example, fenugreek sprouts contaminated with *E. coli* caused 54 deaths in Germany in 2011 (Havelaar et al. 2015). Food preservation aim is to extend the shelf life of food by preventing the growth of bacteria or fungi and stopping biochemical processes in food (Sridhar et al. 2021). Food preservatives can be divided into two types, natural preservatives, like salt, sugar, honey and wood smokes and synthetically manufactured food Preservatives. The common chemical antimicrobial preservatives include propionate, benzoate, nitrite, sulfites and disodium EDTA (Shaker et al. 2022; Russell 1991; Dalton 2002; Ng et al. 2019). Biological control by antagonists such as bacteria can biologically out-compete toxigenic fungi or decrease their mycotoxin levels (Medeiros et al. 2012).

Chemicals used in food preservation (traditional preservatives), despite being the most widely used in the world, as they extend the shelf life of foods and prevent the growth of pathogens in addition to improving flavor and color, they have many side effects and have a significant impact on humans' health (Marrez et al. 2017; Embaby et al. 2019). Diseases, such as headache, lethargy, and hyperactivity of the immune system, may cause cancer as a result of the long-term use of preservatives (Olofinnade et al. 2021). Nitrates and nitrites are added to meat to improve flavor, maintain the red color and control microbial growth, but it has shown that they can lead to produce nitrosamines, which are classified by the International Agency for Research on Cancer (IARC) as carcinogens (Coviello et al. 2020). Benzoate is used widely to preserve many food products, such as juices and soft drinks, and is classified as GRAS, but there are some diseases, such as memory loss, anxiety and motor impairment, that are attributed to the consumption of beverages containing benzoate (Olofinnade et al. 2021). Food additives have a significant impact on children and cause tantrums and disruptive behavior (Baudouin et al. 2010; Dwivedi et al. 2017).

Recently, plant extracts such as polyphenols have been used in food preservation (Sridhar et al. 2021). Therefore, one of the most recent choices is to use natural food preservative (such as plant extracts) or use several combination systems between synthetically food preservatives and natural extracts. This may minimize or prevent the side effects of these chemical compounds and improve the synergistic effect of these synthetic compounds and natural extracts against foodborne microorganisms (Marrez et al. 2017; Embaby et al. 2019). *Alhagi graecorum* Boiss. is a plant commonly found in Egypt, belongs to the family *Fabaceae* (Elsaied et al. 2018). It grows naturally in the dry, rocky, wet environments and salty soils of the eastern and western deserts, the Mediterranean, the Red Sea coast and the land of the Nile. It is an evergreen herb covered with thorny twigs (Salama et al. 2021), with hemicryptophytes life form and perennial life span (Nafea 2019). The most common names of *Alhagi graecorum* Boiss. in Egypt are “AL-Agool” (Kandal and Hassan 2020) and “camel thorn” (Abd El-hak et al. 2019). There are 12 different types of flavonoids that were isolated and identified from *Alhagi graecorum* Boiss. (Ahmad et al. 2015), as well as several other polyphenolic compounds, such as alkaloids, phenols, terpenoids, resins and other secondary metabolites. *A. graecorum* possess many biological activities such as antimicrobial, antioxidant, anti-proliferative and cytotoxic properties. Accordingly, it enters into many medicinal uses (Salama et al. 2021; Saleh and Madany 2014). Numerous studies have shown the use of *Alhagi* species in treatment of some diseases, such as gastroenteritis, ulcers, fever and cancer (Abd El-hak et al. 2019). *A. graecorum* extracts are used to heal constipation and hemorrhoids, in addition, being used as an analgesic for chronic migraines (Al-Edany 2021). It is also useful for rheumatism, infections of the liver and urinary tract, and it is used as a laxative and a schistosomiasis repellent (Muhammad et al. 2015). Al-Massarani and El Dib (2015) reported that *A. graecorum* possess anti-bacterial, anti-fungal and anti-cancer effect.

Find and develop new antimicrobial approaches has become an urgent necessity. The combination between traditional food preservatives and plant extracts may be one of these techniques (Shi et al. 2017). Therefore, this study was conducted to evaluate the anti-bacterial activity and the potential synergistic effect of the ethanolic extract of *A. graecorum* with two conventional food preservatives against 5 foodborne bacterial strains, supported by HPLC assay and molecular docking modeling of the higher concentration compounds. This may enhance the use of natural anti-bacterial agents and reduce the harmful traditional preservatives use.

## Materials and methods

### Sample collection and preparation

The aerial parts of camel thorn *Alhagi graecorum* Boiss. were collected from the garden of National Research Centre, Cairo, Egypt. Plant materials were identified by Dr. Abdelhalem Mohamed (Flora and Plant Classification Research Department, Horticultural Research institute, ARC, Egypt). A voucher specimen was deposited at the herbarium of Agriculture Research Center with number M339. The plant parts washed with distilled water and dried in National Research Centre Dokki, Cairo, Egypt using solar dryer. The dried parts were ground using Braun Multiquick Mixer (4250 Original, Germany).

### Extract preparation

The successive extraction technique was performed for 100 g using four solvents, hexane, diethyl ether (anhydrous), chloroform and ethanol with continuous mixing in a reciprocating shaker (MP-7552, hsiHefer, San Francisco). The residual was separated by filtration and the filtrate of each extract was dried using rotary evaporator (Heidoph, North America) at 50 °C. As the ethanol extract presented higher anti-bacterial activity, it was selected to study the synergistic effect.

### Phenolic profile determination using HPLC

The phenolic profile of *Alhagi graecorum* ethanolic extract was determined using High-Performance Liquid Chromatography (HPLC). HPLC analysis was carried out according to Kim et al. (2006) using Agilent Technologies 1260 series liquid chromatograph equipped with an auto-sampler and a diode-array detector. The separation was carried out using Eclipse XDB-C18 (4.6 mm × 250 mm i.d., 5 μm) with a C18 guard column (Phenomenex, Torrance, CA). The mobile phase consisted of water (A) and 0.05% tri-fluoro-acetic acid in acetonitrile (B) at a flow rate 1 mL/min. The mobile phase was programmed consecutively in a linear gradient as follows: 0 min (82% A); 0–5 min (80% A); 5–8 min (60% A); 8–12 min (60% A); 12–15 min (82% A) and 15–16 min (82% A). The multi-wavelength detector was monitored simultaneously at 280, 320, and 360 nm. The injection volume was 10 μL for each of the sample solutions and peaks were monitored simultaneously at 280, 320, and 360 nm. The column temperature was set during the separation process to 35 °C.

### Bacterial strains

The inhibitory effect of *Alhagi graecorum* ethanolic extract was performed on five strains of foodborne pathogenic bacteria, two Gram-positive bacteria: *Bacillus cereus* EMCC 1080, *Staphylococcus aureus* ATCC 13565 and three Gram-negative bacteria: *Salmonella typhi* ATCC 25566, *Escherichia coli* 0157 H7 ATCC 51659, and *Pseudomonas aeruginosa* NRRL B-272. The stock cultures were grown on nutrient agar slant at 37 °C for 24 h and then kept in refrigerator till use.

### Disk diffusion assay

*Alhagi graecorum* ethanolic extract, sodium benzoate, and sodium propionate were tested for anti-bacterial activity using disk diffusion method as described by Bauer (1966) and Marrez et al. (2019). A loop full of bacteria, incubated for 24 h in a nutrient agar slant of each bacterial species, was inoculated in a test tube containing 5 mL of Mueller–Hinton broth. Broth culture was incubated at 37 °C for 4 h until it achieved turbidity of 0.5 McFarland BaSO_4_ standard (10^8^ CFU mL^−1^), then spread on Mueller–Hinton agar with a sterile cotton swab. Sterile filter paper disks (6 mm diameter) were loaded by extracts (10 µg mL^−1^) and were placed on top of the culture. DMSO represented the negative control and ceftriaxone (500 μg mL^−1^) was used as a positive control. After that, inoculated plates were incubated at 37 °C for 24 h. The plates were incubated at 37 °C for 24 h. Evidence of clear zone indicates bacterial growth inhibition and the diameters were measured in mm.

### Determination of minimum inhibitory concentration (MIC)

Determination of minimal inhibitory concentration (MIC) for *Alhagi graecorum* ethanolic extract, sodium benzoate, and sodium propionate was performed using the microbroth dilution method according to Andrews (2001). Twofold serial dilutions of the ethanolic extract, sodium benzoate, and sodium propionate ranging from 5 to 0.1 mg mL^−1^ were used. Equal volumes of tested bacteria (10^5^ CFU/mL) were added to each well. The MIC was defined as the lowest concentration of antimicrobial agent that was able to inhibit the bacterial growth after 24 h of incubation at 37 °C.

### Checkerboard assay

The presence of synergism, additive or antagonism of *Alhagi graecorum* ethanolic extract with sodium benzoate, and sodium propionate was evaluated using isobolograph analyses and the checkerboard assay according to Tallarida (2001). This method was conducted using different concentrations of the ethanolic extract and sodium benzoate or sodium propionate along different axes, ensuring that each well contained different combinations of the ethanolic extract and sodium benzoate or sodium propionate. The analyses were performed using 96-well plates. Bacteria were grown to reach 2 × 10^8^ CFU mL^−1^. Five microliters of each bacterial strain inoculum was added into the well containing tested ethanolic extract and sodium benzoate or sodium propionate and Mueller–Hinton Broth medium (MHB). The plates were incubated for 18 h/37 °C.

MIC was determined for the combination as the lowest concentration that completely inhibited bacterial growth. Fractional inhibitory concentration (FIC) was calculated for each combination using the following formula: FICA = MICA in combination/MICA alone; FICB = MICB in combination/MICB alone; FIC index = FICA + FICB, where MICA is the MIC of sodium benzoate or sodium propionate, FICA is the FIC of sodium benzoate or sodium propionate, MICB is the MIC of the ethanolic extract, and FICB is the FIC of the ethanolic extract. FIC index is the FIC added value of both sodium benzoate or sodium propionate and the ethanolic extract. The interaction of the anti-bacterial combinations was determined as previously reported by Bansal et al. (2010) and Mandalari et al. (2010) by plotting an isobologram.

### Time-kill curve assay

Time-kill curves were assayed using the confirmed synergistic combinations of *Alhagi graecorum* ethanolic extract with sodium benzoate, and sodium propionate against the selected foodborne pathogenic bacteria. The overnight growth plate was inoculated in sterile MHB at 35 °C to approximate the density of 0.5 McFarland standard. The suspension was diluted 1:10 in normal saline solution to obtain a standard inoculum of 1 × 10^6^ CFU mL^−1^. An amount of 100 µL of the diluted bacterial suspension was added to 0.9 mL of MHB. Double dilutions for each ethanolic extract and sodium benzoate or sodium propionate were prepared. Tubes containing the synergistic combination were incubated at 35 °C for 24 h. From each tube, 100 µL of the sample was collected at 0, 4, 8, 12, and 24 h and plated to determine the count of viable cells. Additionally, growth control was included for each assay. The killing rate was determined by plotting colony viable counts (CFU/mL) against time. Synergy was defined as a ≥ 2 log10 CFU mL^−1^ reduction in viable bacteria with the combination compared with the most active single agent.

### Molecular docking

Molecular docking was carried out—using Molecular Operating Environment molecular (MOE^®^) version 2014.09 (Chemical Computing Group Inc., Montreal, Canada)—with five selected phenolic compounds (gallic acid, chlorogenic acid, ellagic acid, ferulic, and rutin) into the active site of Topoisomerase ATPase enzyme.

Two-dimensional structures of the tested compounds were drawn on MOE software using smile codes, which is downloaded from the National library of Medicine (https://pubchem.ncbi.nlm.nih.gov/). The molecules were prepared for docking using ligand preparation protocol, the 3D structures were protonated and the energies were minimized. Finally, the molecules were saved as MOE molecule file. The X-ray crystallographic structure of DNA gyrase in complex with 07N as inhibitor (PDB ID: 3TTZ) was downloaded from the protein data bank (https://www.rcsb.org/). The enzyme was prepared for the docking study by removing of the redundant chains, water molecules, solvent molecules and the ligands that are not involved in the binding. Then the enzyme was prepared using protonate 3D protocol in MOE software with the default options. Docking setup was validated by re-docking of the co-crystallized ligand 07N with the enzyme. The re-docked setup resulted in affinity value =  − 7.4 kcal/mole and RMSD of 0.4. The validated setup was then used for prediction of the binding mode and interactions between the compounds and the enzyme. Binding degree of the compounds to the protein (between the compounds and the amino acids) was determined and judged using internal energy scores, bond type and lengths, which is restricted to ≤ 3.5 Å (Mashat et al. 2019; Althagafi et al. 2019).

### Statistical analysis

The Web Agri Stat Package (WASP)—at ICAR (Central Coastal Agricultural Research Institute) was used for the statistical analysis. The results were subjected to one-way analysis of variance (ANOVA) to analyze the difference between groups by applying the critical difference. All tests were treated in three replicates (*p* < 0.05).

## Results

### Phenolic profile of *Alhagi graecorum* ethanolic extract

**Fig. 1:**
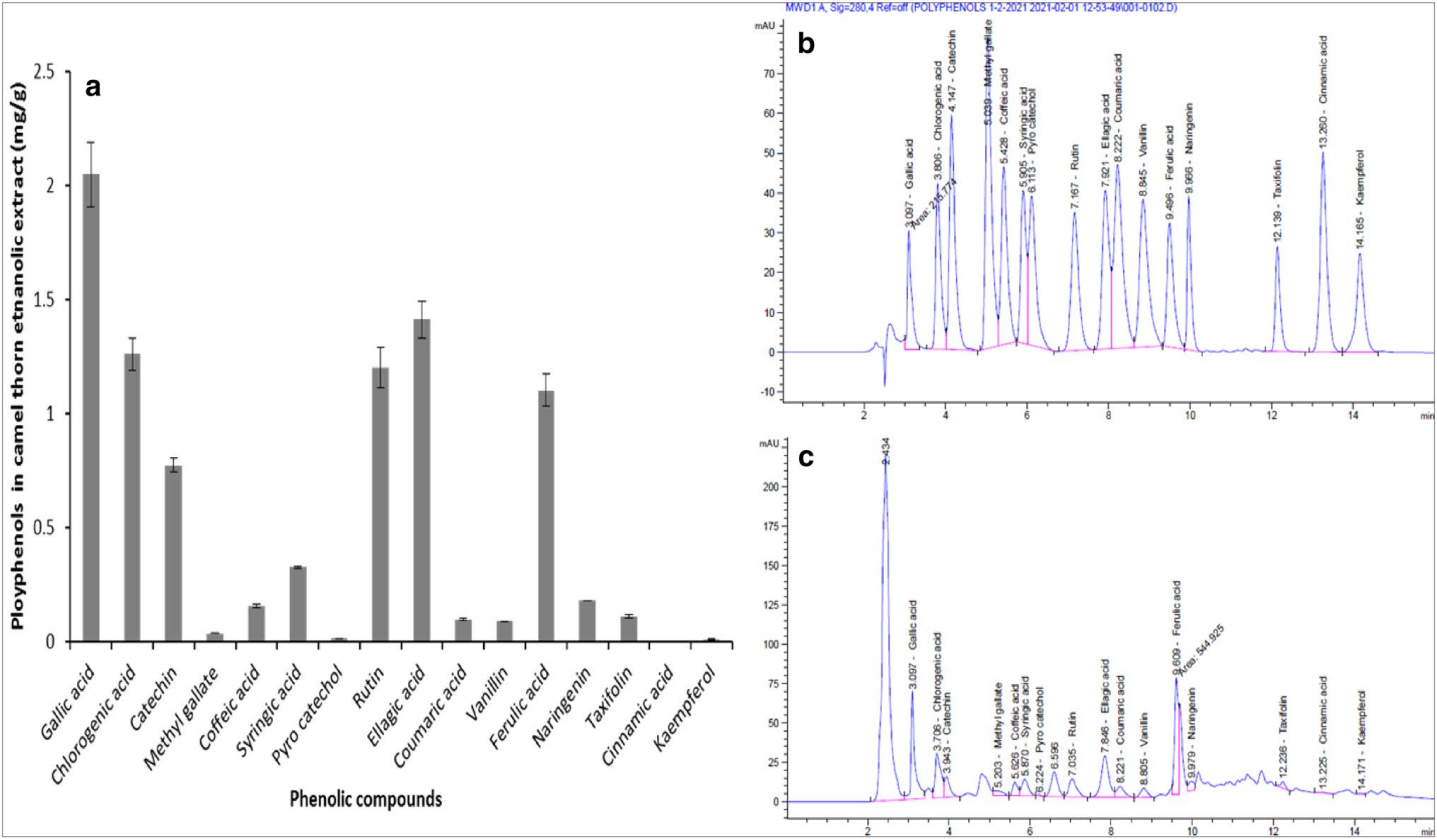
Phenolic profile of *Alhagi graecorum* Boiss. Ethanolic extract, **a** concentration of polyphenols (mg/g); **b** HPLC chromatogram of polyphenols standards; **c** HPLC chromatogram of ethanolic extracts of *Alhagi graecorum* Boiss

Figure 1 illustrates the polyphenolic compounds present in *Alhagi graecorum* Boiss. ethanolic extract that identified and quantified using HPLC. Sixteen phenolic compounds were found in *A*. *graecorum* ethanolic extract. Gallic acid was recorded as the highest phenolic compound with concentration of 2.05 mg g^−1^ extract, followed by ellagic acid, chlorogenic acid, rutin, ferulic acid and catechin with concentrations of 1.41, 1.26, 1.20, 1.10 and 0.77 mg g^−1^, respectively. While, the lowest concentration of phenolic compound (0.004 mg g^−1^) was recorded by cinnamic acid, followed by kaempferol, pyro-catechol and methyl gallate with concentrations of 0.012, 0.02 and 0.04 mg g^−1^, respectively.

### Antibacterial activity of *A. graecorum* ethanolic extract

As shown in Table 1, the anti-bacterial activity of camel thorn *A. graecorum* ethanolic extract and two conventional food preservatives, sodium benzoate and sodium propionate against two Gram-positive and three Gram-negative foodborne pathogenic bacteria were determined. DMSO was represented as negative control and ceftriaxone as positive control. The ethanolic extract showed anti-bacterial activity against all tested bacteria, the highest activity was shown against *Staph. aureus*, *E. coli* and *P. aeruginosa* with inhibition zone values 9.3, 9.2 and 9 mm, respectively. While, the lowest inhibition zones 7.5 and 7.8 mm were recorded against *S. typhi* and *B. cereus*, respectively. The ethanolic extract had higher anti-bacterial activity than both sodium benzoate and sodium propionate against *P. aeruginosa*, while these conventional food preservatives outperformed the ethanolic extract against the other tested bacteria.

**Table 1:** Antibacterial activity of *A. graecorum* ethanolic extract against some foodborne pathogenic bacteria

Bacteria	Inhibition zone (mm) (Mean ± SE)				
	−Ve control	Ethanol extract	Na-benzoate	Na-propionate	+Ve control
*B. cereus*	0	7.8 ± 1.7^b^	11.0 ± 1.47^a^	10.5 ± 1.08^a^	10.7 ± 0.17^a^
*Staph. aureus*	0	9.3 ± 0.60^c^	9.5 ± 0.71^c^	10.3 ± 0.65^b^	22.3 ± 0.73^a^
*E. coli*	0	9.2 ± 0.17^c^	9.5 ± 1.32^c^	10.6 ± 1.26^b^	15.5 ± 0.76^a^
*S. typhi*	0	7.5 ± 0.29^c^	9.8 ± 1.04^b^	11.6 ± 2.28^a^	10.5 ± 0.29^ab^
*P. aeruginosa*	0	9 ± 0.5^b^	9.2 ± 0.41^c^	8.9 ± 0.48^c^	11.0 ± 1.00^a^

*n* = 3, SE: standard error, different superscripts within row are significantly different at 5% level, critical difference (0.05) = 1.220, negative control: DMSO, positive control: ceftriaxone

### MIC and synergy interaction of *A. graecorum* ethanolic extract with Na-benzoate and Na-propionate

The anti-bacterial activities of *A. graecorum* ethanolic extract against five strains of foodborne bacteria alone and in combination with Na-benzoate are summarized in Table 2. Both ethanolic extract and Na-benzoate showed different anti-bacterial activities against the tested bacterial strains based on the MIC values. The MICs of the ethanolic extract against the tested strains ranged from 1.2 to 1.8 mg mL^−1^, and ranged from 0.6 to 0.9 mg mL^−1^ with Na-benzoate. The FICI values were calculated to observe the synergistic interaction. Strong synergistic effect for the combination of ethanolic extract and Na-benzoate was observed against *B. cereus*, *Staph. aureus* and *S. typhi* with FICI values 0.31, 0.31 and 0.38, respectively. While, this combination showed additive interaction against *E. coli* and *P. aeruginosa* with FICI value of 0.56 and 0.75, respectively.

**Table 2:** Synergic interaction between ethanol extract of *A. graecorum* and sodium benzoate against the tested foodborne pathogenic bacteria

Bacteria	MIC (mg mL^−1^)		FICB	FICE	FIC Index	Interaction
	MICB	MICE				
*B. cereus*	0.9 ± 0.06	1.2 ± 0.2	0.25	0.06	0.31	S
*Staph. aureus*	0.6 ± 0.11	1.2 ± 0.2	0.06	0.25	0.31	S
*P. aeruginosa*	0.6 ± 0.06	1.8 ± 0.6	0.50	0.25	0.75	A
*E. coli*	0.6 ± 0.0	1.8 ± 0.6	0.06	0.50	0.56	A
*S. typhi*	0.6 ± 0.1	1.2 ± 0.2	0.13	0.25	0.38	S

*n* = 3, MICB: minimum inhibitory concentration of sodium benzoate; MICE: minimum inhibitory concentration of ethanol extract; FICB: fractional inhibitory concentration of sodium benzoate; FICE: fractional inhibitory concentration of ethanol extract; S: synergistic effect; A: additive effect; the combination defined synergy if A: additive effect; the combination defined synergy if ƩFIC Index ≤ 0.5 and additive if ƩFIC Index 0.5–1, antagonism if ƩFIC Index > 4. MICs’s values are expressed as mean ± SD

As shown in Table 3, the MIC of Na-propionate against the tested foodborne pathogenic bacteria was ranged between 0.5 and 0.9 mg mL^−1^. Significant decrease in MICs value of Na-propionate (from 50 to 94%) was observed due to the combination with *A. graecorum* ethanolic extract; this reduction in MICs values depended upon the type of bacterial strain. Also, there was significant decrease in the MICs of the ethanolic extract when combined with Na-propionate. The interaction between the ethanolic extract and Na-propionate had either synergistic or additive effects and no antagonistic effect was recorded. The ethanolic extract enhanced the activity of Na-propionate against *Staph. aureus*, *E. coli*, *S. typhi* and *P. aeruginosa* as a synergistic interaction with FICI values 0.31, 0.31, 0.37 and 0.38, respectively. While, additive effect was shown against *B. cereus* with FICI value 0.63.

**Table 3:** Synergic interaction between ethanol extract of *Alhagi graecorum* and sodium propionate against the tested foodborne pathogenic bacteria

Bacteria	MIC (mg mL^−1^)		FICP	FICE	FIC Index	Interaction
	MICP	MICE				
*B. cereus*	0.9 ± 0.06	1.2 ± 0.2	0.50	0.13	0.63	A
*Staph. aureus*	0.5 ± 0.06	1.2 ± 0.2	0.06	0.25	0.31	S
*P. aeruginosa*	0.5 ± 0.06	1.8 ± 0.6	0.13	0.25	0.38	S
*E. coli*	0.5 ± 0.06	1.8 ± 0.6	0.06	0.25	0.31	S
*S. typhi*	0.5 ± 0.11	1.2 ± 0.2	0.25	0.13	0.37	S

*n* = 3, MICP: minimum inhibitory concentration of sodium propionate; MICE: minimum inhibitory concentration of ethanol extract; FICP: fractional inhibitory concentration of sodium propionate; FICE fractional inhibitory concentration of ethanol extract; S: synergistic effect; A: additive effect; the combination defined synergy if ƩFIC Index ≤ 0.5 and additive if ƩFIC Index 0.5–1, antagonism if ƩFIC Index > 4. MICs’s values are expressed as mean ± SD

### Time-kill curve assay

**Fig. 2:**
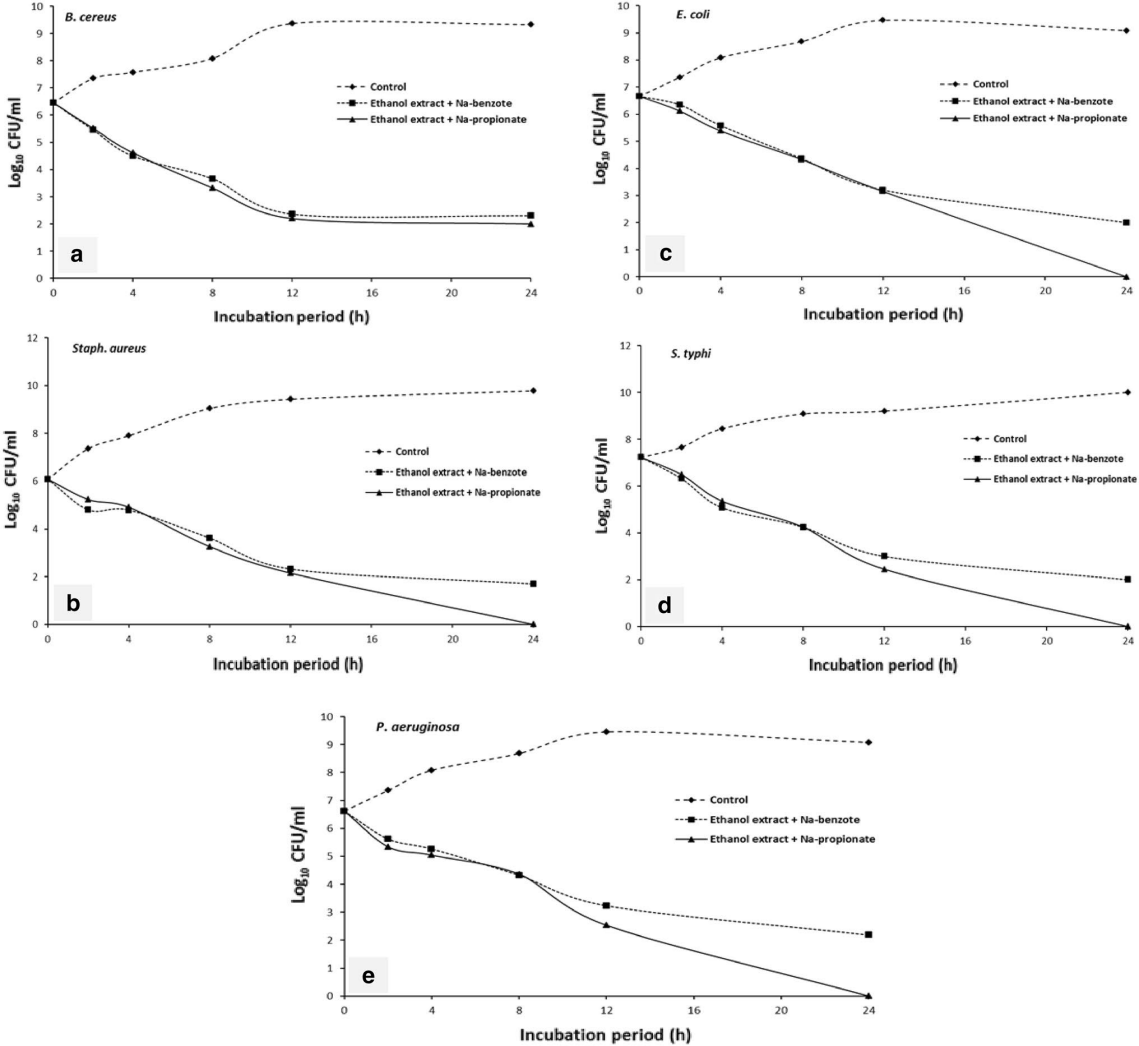
Time-killing data for synergistic combination of *Alhagi graecorum* ethanolic extract with sodium benzote and sodium propionate against, **a** *B. cereus*; **b** *Staph. aureus*; **c** *E. coli*; **d** *S. typhi* and **e** *P. aeruginosa*

Time-kill assay was conducted to confirm the synergistic interaction between *A. graecorum* ethanolic extract and sodium benzoate or sodium propionate against some foodborne pathogenic bacteria as shown in Fig. 2. Time-kill curve assay showed that the synergistic combination of *A. graecorum* extract and sodium benzoate can decrease the growth of *B. cereus*, *Staph. aureus* and *S. typhi* to less than 2.3 log_10_ CFU/mL after 24 h of incubation, while the additive interaction between ethanolic extract and sodium benzoate observed reduction in the population of *P. aeruginosa* and *E. coli* < 2.2 log_10_ CFU/mL after 24 h of incubation. Besides that, the synergistic interaction between the ethanolic extract and sodium propionate showed completely reduction in the growth of *Staph. aureus*, *P. aeruginosa*, *E. coli* and *S. typhi* after 24 h of incubation, whereas the additive combination had decrease the growth of *B. cereus* from 9.32 to 2 log_10_ CFU/mL after 24 h of incubation.

### Molecular docking analysis

Molecular docking was carried out to obtain further information about the binding modes of highly concentrated compounds in the ethanolic extract of *A. graecorum* with the active site of DNA Topoisomerase II. Molecular docking simulation study of gallic acid, chlorogenic acid, ellagic acid, ferulic, and rutin was performed to predict the anti-bacterial activity of these compounds. Furthermore, to understand the different binding modes and the interactions between these compounds and the active site of DNA gyrase, the results of docking’s scores ranged from − 4.2 to − 7.29 kcal\mol comparing to 07N inhibitor as a reference drug − 7.4 kcal\mol. Rutin possesses the highest binding affinities, while chlorogenic acid, ellagic acid and ferulic showed medium binding affinities, whereas gallic acid showed the lowest binding affinities to Topoisomerase ATPase enzyme (Table 4).

**Table 4:** Docking analysis of the active site of Topoisomerase ATPase enzyme (energy scores (S), distances and interactions) for the original ligand and the tested compounds

No.	Compound	S (energy score)	Interaction	Distance Å
1	Original ligand	− 7.4	(ASP 81) H-donor	2.76 Å
			(ARG 144) H-acceptor	2.74 Å
2	Gallic acid	− 4.2	(ASP 81) H-donor	3.21 Å
3	Chlorogenic acid	− 5.74	(ASP 81) H-donor	3.22 Å
			(ARG 144) H-acceptor	3.02 Å
			(ARG 144) H-acceptor	3.33 Å
4	Ellagic acid	− 5.69	(ASP 81) H-donor	3.07 Å
			(GLY 85) H-acceptor	3.18
5	Ferulic	− 5.26	(ASP 81) H-donor	3.38 Å
			(ARG 144) H-acceptor	2.99 Å
6	Rutin	− 7.29	(ASP 81) H-donor	3.03 Å
			(ARG 84) H-acceptor	3.12

**Fig. 3:**
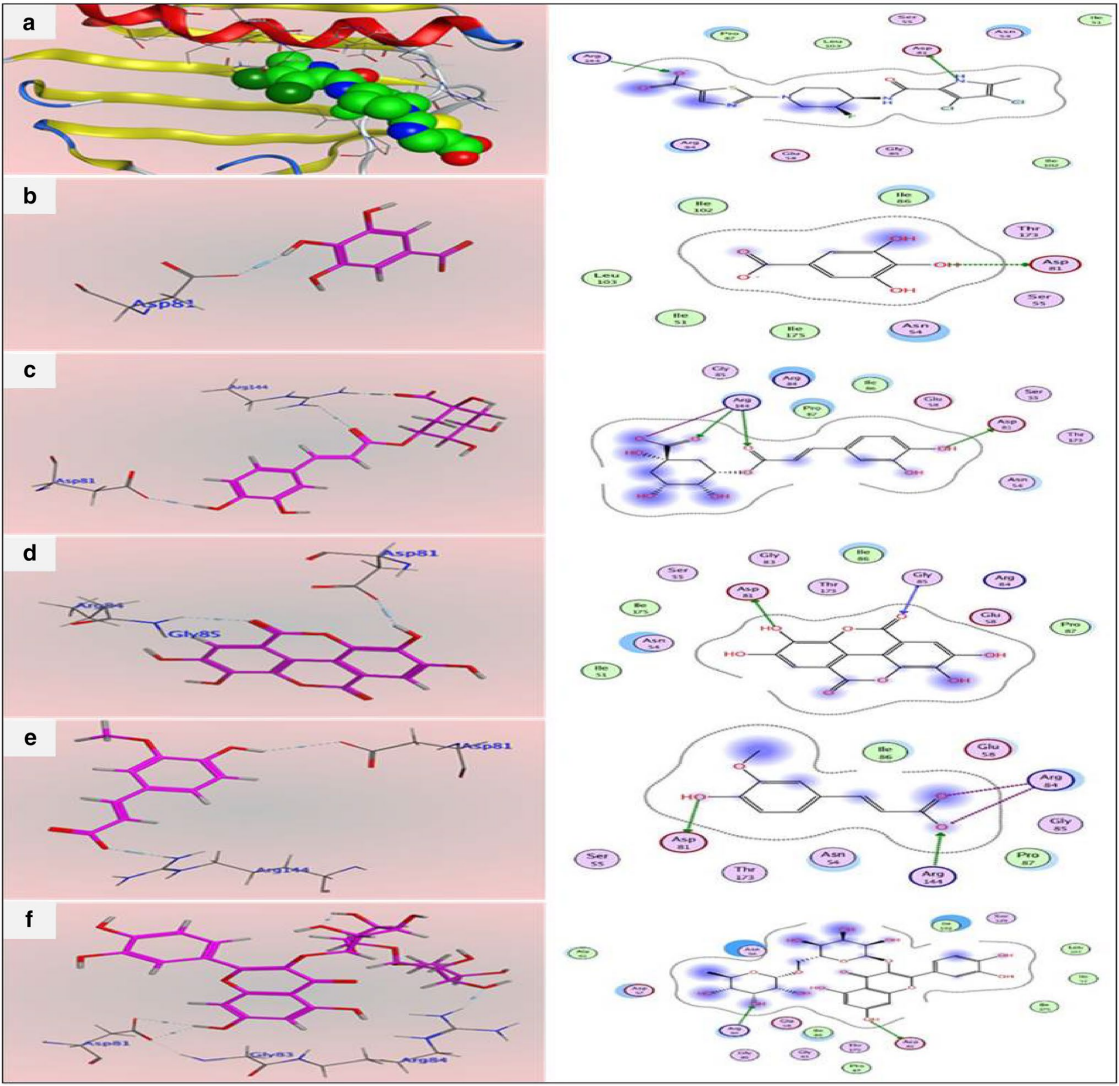
Predicted interactions and binding patterns with the active site of Topoisomerase ATPase (3TTZ); **a** Ligand 07N (green) 3D and 2D interaction pattern with Topoisomerase ATPase (3TTZ), **b** the predictive binding mode (3D and 2D) of gallic acid with Topoisomerase ATPase, **c** the predictive binding mode (3D and 2D) of chlorogenic acid with Topoisomerase ATPase, **d** the predictive binding mode (3D and 2D) of ellagic acid with Topoisomerase ATPase, **e** the predictive binding mode (3D and 2D) of ferulic acid with Topoisomerase ATPase, **f** the predictive binding mode (3D and 2D) of rutin acid with Topoisomerase ATPase

The interaction of the original ligand 07N with the active site of topo-isomerase ATPase has been studied and displayed in 2D and 3D style, presented in Fig. 3a. 07N mediated two H-bond interactions to bind with the key hot spot Asp81 and Arg144 with a distance of 2.76 Å and 2.74 Å respectively. The proposed binding mode of gallic acid (affinity value of − 4.2 kcal/mol), presented in Fig. 3b, showed that, gallic acid has one H-bond interaction with Asp81 via hydroxyl group with a distance of 3.21 Å. The proposed binding mode of chlorogenic acid (affinity value of − 5.74 kcal/mol), presented in Fig. 3c, showed that, chlorogenic acid has one H-bond interaction with Asp81 via hydroxyl group with a distance of 3.22 Å, in addition to mediating two H-bond interactions with Arg144 via carboxyl groups with distances of 3.02 and 3.33 Å. The proposed binding mode of ellagic acid (affinity value of − 5.69 kcal/mol), presented in Fig. 3d, showed that, ellagic acid has two H-bond interactions with Asp 81 and Gly 85 via hydroxyl group with a distance of 3.07 and 3.18 Å, respectively. The proposed binding mode of ferulic acid (affinity value of − 5.26 kcal/mol), presented in Fig. 3e, showed that, ferulic acid has two H-bond interactions with Asp81 and Arg144 via carboxyl group with a distance of 3.38 Å and 2.99 Å respectively. The proposed binding mode of rutin (affinity value of − 7.29 kcal/mol), presented in Fig. 3f, showed that, rutin has two H-bond interactions with Asp81 and Arg84 via hydroxyl group with a distance of 3.03 Å and 3.12 respectively.

## Discussion

HPLC analysis of *A. graecorum* ethanolic extract revealed sixteen phenolic compounds illustrated in Fig. 1. Muhammad et al. (2015) reported that *Alhagi* is a rich source of biologically active phytochemical compounds, such as phenolic compounds, flavonoids, polysaccharides and alkaloids. Al-Jaber et al. (2011) found that the bioactive compounds in *A. maurorum* Boiss. were mainly flavonoids. Ahmed (2019) reported that the aqueous extract of *A. maurorum* had high content of caffeic acid, vanillic acid, naringenin, syringic, chlorogenic, coumaric, ferulic, sinapic acid and rutin. Al-Snafi et al. (2019) revealed that *A. maurorum* phenolic extract had several bioactive compounds which possess anti-bacterial and anti-fungal activities.

Benzoate and propionate are known as conventional food preservatives and are used to prevent the growth of bacteria and fungi in foods as they have anti-fungal and anti-bacterial effects (Glass et al. 2007). Among the plant extracts, *Alhagi* spp. extracts possess several biological activities, such as anti-bacterial, anti-cancer, antioxidant and other effects (Wagay et al. 2018). Zain et al. (2012) indicted that the ethanol extract of *A. maurorum* showed anti-bacterial activity against Gram-negative bacteria: *E. coli*, *Proteus mirabilis*, *P. aeruginosa*, *S. typhi* and Gram-positive bacteria: *B. subtilis*, *Micrococcus luteus*, *Staph*. *aureus*, *Staph*. *haemolyticus* and *Streptococcus pyogenes*. Also, Ahmed et al. (2015) found that *A. maurorum* ethanolic extract had anti-bacterial activity against *Agrobacterium tumefaciens*, *Pseudomonas solanacearum* and *Corynebacterium fascians*. Accordingly, our results corroborate previous studies, where the ethanolic extract of *A. graecorum* and the food preservatives (Na-benzoate and Na-propionate) showed anti-bacterial activity against all tested bacteria.

The combination of the ethanolic extract and Na-benzoate or Na-propionate had synergistic and additive effects against the tested bacterial strains, and the values of MICs were significantly decreased. Stanojevic et al. (2010) reported that the combination of *M*. *officinalis* ethanolic extract with sodium nitrite, sodium benzoate and potassium sorbate showed synergistic interaction against *A*. *tumefaciens*, *B*. *mycoides*, *B*. *subtilis*, *E*. *carotovora*, *E*. *coli*, and *P*. *fluorescens*. Jebelli Javan et al. (2019) revealed the combined effect of *T. ammi* essential oil and ethanolic extract of propolis enhancing the anti-bacterial efficacy against *B*. *cereus*, *Staph*. *aureus*, *E*. *coli*, *S*. *Typhimurium*, and *L. monocytogenes*. Ekhtelat et al. (2020) indicated that the combination of *C*. *cyminum*, *M*. *longifolia* and *M*. *spicata* essential oils with sodium benzoate showed significant decrease in MIC values against *Staph. aureus* and *Y*. *enterocolitica* when compared with essential oils or sodium benzoate. Also, Attia et al. (2021) reported that the combination between the mandarin phenolic extract and the food preservative sodium nitrite showed synergistic anti-bacterial activities against foodborne pathogens: *B. cereus*, *Staph. aureus*, *E. coli*, and *P. aeruginosa*. Embaby et al. (2019) found that *Ficus nitida* phenolic extract had synergistic effect with tetracycline against *B. cereus*, *Staph. aureus*, *E. coli*, *P. aeruginosa* and additive interaction against *S*. *typhi* and *K*. *pneumoniae*, and this result was supported with efflux pump inhibitory activity.

Time-killing curve studies are characterized by giving information about the time course of bacterial activity in contrast to MIC and checkerboard assay (Hacioglu et al. 2017). Hence, the present study used time-killing curve studies, and according to its results, the synergistic interactions between ethanol extract with sodium benzoate or sodium propionate against the tested bacterial strains were just as frequent as those in the checkerboard assay results. The combination of the ethanol extract with sodium propionate showed complete bacterial inhibition after 24 h of incubation, while combination of the ethanol extract with sodium benzoate showed effective decrease in the growth of tested bacteria. Attia et al. (2021) reported that the synergistic combination between mandarin peel extract and NaNO_2_ completely inhibited the growth of *P*. *aeruginosa* and *Staph*. *aureus* within 12 and 24 h of incubation, respectively. While, the synergistic combination between mandarin peel extract and NaNO_2_ observed effective decrease in *B*. *cereus* and *E*. *coli* population ranged from 6 to 7 log cycle CFU/mL. Shi et al. (2017) demonstrated that the combination between nisin and *p*-Anisaldehyde showed a stronger bactericidal activity against *Staph*. *aureus*.

Based on the results obtained from the in vitro study, it was believed that it was worthy to carry out molecular docking study, hence testing the compounds, instilling the results in silico and in vitro. Oyedemi et al. (2020) reported that the methanol extracts of *Ligustrum lucidum* and *Lobelia inflata* showed significant inhibition of DNA gyrase A from *Staphylococcus aureus*, in particular, lubilanidin which showed a similar binding mode such as the original ligand. H-bond interactions between benzothiazole and naphthalene with dihydroorotase (at sites LEU222 or ASN44) led to inhibition of *E. coli* dihydroorotase enzyme, and this may contribute to the antimicrobial effect of these compounds (Morsy et al. 2020). Topoisomerase II is an attractive target for the development of new anti-bacterial agents as it plays a key role in stabilizing the topological state of the DNA (Khan et al. 2018; Orritt et al. 2021). We found that, the compounds with higher concentrations presented in the ethanolic extract of *A. graecorum* interact with the DNA Topo ll in a position and orientation similar to the original inhibitor ligand (07N). The docking study revealed that all tested compounds possessed H-bond interactions with ASP 81 amino acid, similar to the original ligand, and two of the compounds (chlorogenic acid and ferulic acid) interact with ARG 144 amino acid with a hydrogen bond, similar to the original ligand, while three compounds (gallic acid, ellagic acid, and rutin) interacted with different positions (amino acids) from the original ligand. Thus, these obtained interactions explain and confirm the in vitro results of the ethanolic extract of the *Alhagi graecorum* Boiss.

## Conclusion

The results of the present study indicate that camel thorn ethanolic extract has noticeable anti-bacterial activity alone and increased by combination with food preservatives. The combinations of *A. graecorum* extract with Na-benzoate and Na-propionate resulted in both synergistic and additive interactions, and no antagonistic interactions were observed. In silico molecular docking study on DNA gyrase topoisomerase II as a potential target for antimicrobial activity confirmed the inhibitory effect of the *A. graecorum* ethanolic extract. Consequently, it enhances the effectiveness of the conventional preservatives used. Future studies should be performed to isolate pure active compounds from *A. graecorum* to apply them in different types of foods as natural preservatives alone or with conventional preservatives.
